# Host Mucin Is Exploited by Pseudomonas aeruginosa To Provide Monosaccharides Required for a Successful Infection

**DOI:** 10.1128/mBio.00060-20

**Published:** 2020-03-03

**Authors:** Casandra L. Hoffman, Jonathan Lalsiamthara, Alejandro Aballay

**Affiliations:** a Molecular Microbiology and Immunology Department, Oregon Health and Sciences University, Portland, Oregon, USA; Massachusetts General Hospital

**Keywords:** *Caenorhabditis elegans*, *Pseudomonas aeruginosa*, bacterial colonization, host-pathogen interactions, infection, innate immunity, lung cells, *muc1*, mucin, *mul-1*

## Abstract

One of the primary functions of the mucosal barrier, found lining epithelial cells, is to serve as a first-line of defense against microbial pathogens. The major structural components of mucus are heavily glycosylated proteins called mucins. Mucins are key components of the innate immune system as they aid in the clearance of pathogens and can decrease pathogen virulence. It has also been recently reported that individual mucins and derived glycans can attenuate the virulence of the human pathogen Pseudomonas aeruginosa. Here, we show data indicating that mucins not only play a role in host defense but that they can also be subverted by P. aeruginosa to cause disease. We found that the mucin MUL-1 and mucin-derived monosaccharides *N*-acetyl-galactosamine and *N*-acetylglucosamine are required for P. aeruginosa killing of Caenorhabditis elegans. We also found that the defective adhesion of P. aeruginosa to human lung alveolar epithelial cells, deficient in the mucin MUC1, can be reversed by the addition of individual monosaccharides. The monosaccharides identified in this study are found in a wide range of organisms where they act as host factors required for bacterial pathogenesis. While mucins in C. elegans lack sialic acid caps, which makes their monosaccharides readily available, they are capped in other species. Pathogens such as P. aeruginosa that lack sialidases may rely on enzymes from other bacteria to utilize mucin-derived monosaccharides.

## Abstract (precis)

One of the first lines of defense present at mucosal epithelial tissues is mucus, which is a highly viscous material formed by mucin glycoproteins. Mucins serve various functions, but importantly they aid in the clearance of pathogens and debris from epithelial barriers and serve as innate immune factors. In this study, we describe a requirement of host monosaccharides, likely derived from host mucins, for the ability of Pseudomonas aeruginosa to colonize the intestine and ultimately cause death in Caenorhabditis elegans. We also demonstrate that monosaccharides alter the ability of bacteria to bind to both Caenorhabditis elegans intestinal cells and human lung alveolar epithelial cells, suggesting that there are conserved mechanisms underlying host-pathogen interactions in a range of organisms. By gaining a better understanding of pathogen-mucin interactions, we can develop better approaches to protect against pathogen infection.

## INTRODUCTION

Epithelial barriers have evolved multiple mechanisms to respond to environmental cues, including those associated with pathogen exposure. The mucus layer found lining epithelial cells is a crucial first barrier against physical damage, dehydration, and infection (1–3). The main structural components of the protective barrier of mucus are mucins, which are high-molecular-weight proteins, heavily glycosylated at serine and threonine residues (1, 2, 4, 5). Mucins are secreted in large quantities by mucosal epithelial cells, and both membrane-tethered and secreted mucins are found on the apical surface of all mucosal epithelia. Mucins form a viscous gel, trap microbes, aid in the clearance of microbes, form a physical barrier, and provide a matrix for a rich array of antimicrobial molecules (1, 2). In addition to these roles, certain mucins and their glycans have been shown to attenuate bacterial virulence; these glycans have been shown to downregulate toxin and siderophore expression, increase biofilm dispersal, and reduce cytotoxicity to epithelial cells (3).

Despite the various defensive properties of mucins, some bacterial pathogens are able to colonize mucosal epithelial barriers (3–7). Microbes must first penetrate the mucosal barrier either to attach to epithelial cells or to release toxins that disrupt the epithelial barrier (2, 7–10). One of the low-affinity binding mechanisms used by microbes is mediated by hydrophobic interactions with lectins and glycosylated receptors, including oligosaccharides and monosaccharides present on mucins (4, 11–13). In addition to serving as a binding site, individual monosaccharides from mucins can be accessed as energy sources by bacteria with mucolytic activity (14). Finally, mucus components can influence the virulence characteristics of pathogenic bacteria, such as virulence factor expression, adhesion, motility, proliferation, and/or growth (3, 7, 8, 15–19).

Alterations in mucin expression or glycosylation patterns have been linked to various pathologies and diseases, including cystic fibrosis, chronic obstructive pulmonary disease, cancers, and inflammatory bowel disease (20). These diseases, which are associated with changes in mucin expression, glycosylation patterns, and alterations in mucus levels, are associated with higher rates of infections by opportunistic pathogens (21–23). Increases in mucin expression often result in a more highly viscous material that is not readily cleared from the mucosal barrier. Decreases in mucus levels remove the protective barrier, which allows pathogens to have direct access to epithelial cells. Many of the diseases and conditions linked to altered mucus levels have no known cure; the primary mode of treatment involves controlling mucin expression, which can also be a key method used to prevent the associated bacterial infections (22, 24). With various sources of data showing that mucins may benefit both the host and the pathogen, our study aims to identify novel bacterium-mucin interactions that favor only the bacteria or only the host. A better understanding of the mechanisms by which pathogens interact with host mucins will provide novel approaches to control infectious processes and prevent bacterial colonization.

The complexity of mammalian epithelial mucosal surfaces and the additional functions of the innate immune system can mask the roles of individual mucins at epithelial barriers. Using the model organism Caenorhabditis elegans, we can tease apart the roles of individual mucins at the intestinal epithelial barrier. This model provides the advantage of a simple organism’s small genome size and the absence of adaptive immunity. C. elegans eats bacteria found in decomposing organic matter (25). Several pathogens are present in the environment in which C. elegans feeds, including Pseudomonas aeruginosa, which can impair the growth of the nematode and induce stress responses, ultimately leading to nematode death (26). In the laboratory, the animals are cultured monoxenically by being fed Escherichia coli. E. coli is effectively disrupted by the C. elegans pharyngeal grinder, and essentially no intact bacteria can be found in the intestinal lumen. However, P. aeruginosa is capable of attaching to the intestinal epithelial cells and colonizing the gut, ultimately killing C. elegans via an infectious process that depends on virulence factors that are required for full pathogenesis, not only in nematodes, but also in mammalian and plant hosts (27). As in all metazoans, the ability to distinguish between pathogenic and nonpathogenic microbes is critical for the survival of infected nematodes. Thus, C. elegans has evolved mechanisms to recognize and counteract pathogens, and in response to P. aeruginosa, the nematode activates several signaling pathways, including the p38 mitogen-activated protein kinase (MAPK) *pmk-1* pathway, the FOXO transcription factor *daf-16*, and the transforming growth factor β (TGF-β) signaling pathway (27–32). Part of the C. elegans defense response against infection also consists of the upregulation of mucins (29, 33). Several C. elegans mucins are significantly increased upon exposure to P. aeruginosa; thus, it would appear that these mucins are important immune factors that play beneficial roles for C. elegans (29, 33).

Here, we found that inhibition by RNA interference (RNAi) or deletion by CRISPR/Cas9 of *mul-1* enhances C. elegans resistance to infection by P. aeruginosa or Salmonella enterica. MUL-1 is an intestinally expressed, secreted mucin and has been implicated in the response to both gamma irradiation and cadmium exposure (34, 35). In addition, expression of *mul-1* has been reported to be controlled by the *pmk-1* p38/MAPK, the *daf-*2 and *daf-16* insulin-like, and the *dbl-1* TGF-β signaling pathways (34). The regulation of *mul-1* by various immune signaling pathways suggests that it is an innate immune factor, involved in a variety of responses to pathogen infection and host damage. In contrast to this suggested role and the typical role of mucins as immune effectors, our results indicate that certain pathogens use MUL-1 to tip the balance in their favor during infection. Our results also indicate that MUL-1 may be a source of monosaccharides that are required for bacterial colonization of the C. elegans intestine and that specific monosaccharides are able to increase toxin production and biofilm formation. We also show that these same monosaccharides aid in attachment to mammalian epithelial cells. These results indicate that P. aeruginosa exploits conserved immune effectors, perhaps even specific monosaccharides derived from mucins, to colonize a range of hosts.

## RESULTS

### C. elegans lacking *mul-1* exhibit enhanced resistance to P. aeruginosa infection.

**FIG 1:**
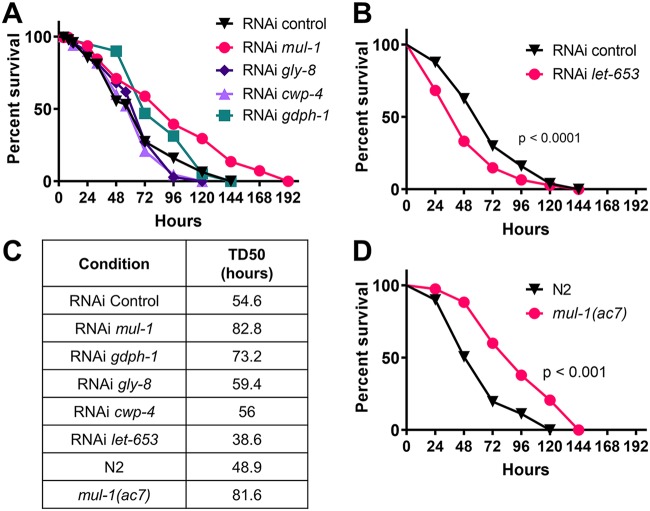
RNAi silencing and deletion of *mul-1* result in an enhanced resistance to P. aeruginosa. (A) Young adult control and RNAi-expressing animals were exposed to P. aeruginosa (3 biological and 3 technical replicates; *n* = 180 animals per condition). (B) Young adult control and *let-653* RNAi animals were exposed to P. aeruginosa (3 biological and 3 technical replicates; *n* = 120 per condition). *P* values for results compared to those with the RNAi control were as follows: *mul-1*, *P* < 0.0001; *let-653*, *P* < 0.001; *gly-8*, *P* = 0.5594; *cwp-4*, *P* = 0.1336; *gpdh-1*, *P* = 0.0079. (C) Time to 50% death (TD_50_) upon nematode exposure to P. aeruginosa was calculated for each of the survival curves shown in panels A and B using GraphPad Prism, version 8. (D) Young adult and *mul-1*(*ac7*) animals were exposed to P. aeruginosa (3 biological and 3 technical replicates; *n* = 180 animals per condition).

Little is known about the roles of specific mucins in the C. elegans intestine during infection, but several enzymes and transporters that modify the glycan patterns of mucins have been identified to alter host-pathogen interactions (36, 37). We set out to better understand the roles of intestinally expressed mucins during C. elegans defense against P. aeruginosa infection. The five identified mucins and mucin-editing enzymes (see Table S1 in the supplemental material) were selected based upon conserved serine- and threonine-rich regions, intestine-expressed mRNA transcripts, and altered expression upon P. aeruginosa infection. The selected genes were silenced by RNAi, and the survival of the animals was monitored during P. aeruginosa infection. RNAi targeting *mul-1* and *gpdh-1* resulted in enhanced resistance to pathogen infection, and RNAi for *let-653* resulted in enhanced susceptibility to pathogen infection (Fig. 1A to C). RNAi targeting the remaining mucins resulted in no changes to C. elegans susceptibility to P. aeruginosa (Fig. 1A to C).

10.1128/mBio.00060-20.7 TABLE S1 List of selected mucin-like and mucin-related genes found in the C. elegans genome. The table includes gene name and sequence, a brief description, and links to infection. Mucin-like and -related genes were identified first in WormBase (https://wormbase.org); those not expressed in the intestine were excluded. Mucin-like genes were validated for the presence of serine- and threonine-rich regions subject to heavy glycosylation. Download Table S1, PDF file, 0.03 MB. Copyright © 2020 Hoffman et al. 2020 Hoffman et al. This content is distributed under the terms of the Creative Commons Attribution 4.0 International license.

Because mucins are upregulated upon infection and have been characterized as bona fide immune effectors, the enhanced resistance phenotype for two of the genes was unexpected. The most significant phenotype was observed for RNAi *mul-1* animals, which showed enhanced resistance to P. aeruginosa compared to that of RNAi control animals (Fig. 1A). To confirm the role of *mul-1* during infection, an in-frame deletion strain was used. The *mul-1*(*ac7*) strain also showed enhanced resistance to P. aeruginosa (Fig. 1D). Expression of *mul-1* is upregulated upon infection with P. aeruginosa at 4 and 8 h (29, 33), which would suggest that *mul-1* is an immune response factor that is turned on to combat pathogen infection. Our results suggest the opposite in the case of P. aeruginosa, i.e., that the mucin is exploited during P. aeruginosa infection to benefit the pathogen.

### The enhanced resistance of RNAi *mul-1* animals to P. aeruginosa is due to reduction of bacterial colonization.

**FIG 2:**
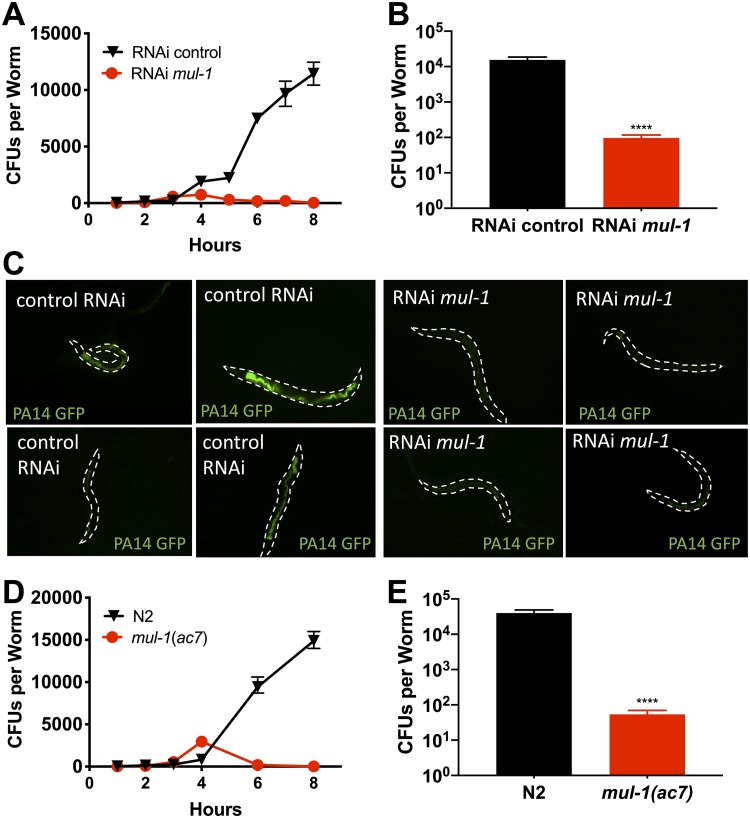
RNAi silencing and deletion of *mul-1* results in reduced P. aeruginosa colonization. (A) Young adult control and *mul-1* RNAi animals were exposed to P. aeruginosa-GFP. At indicated time points, CFU counts/nematode were determined (3 biological and 3 technical replicates; *n* = 90 animals per condition, per time point). (B) CFU counts at 24 h (3 biological and 3 technical replicates; *n* = 90 animals per condition). ****, *P* < 0.001. (C) PA14-GFP bacteria were visualized in infected nematodes at 24 h. Representative images of ∼50 animals per condition from two biological replicates are shown. (D) Young adult wild-type and *mul-1*(*ac7*) animals were exposed to P. aeruginosa PA14-GFP. At indicated time points, CFU counts per nematode were calculated (3 biological and 3 technical replicates; *n* = 90 animals per condition per time point). (E) CFU counts at 24 h (3 biological and 3 technical replicates; *n* = 90 animals per condition). ****, *P* < 0.001. The limit of detection for all CFU calculations was 10^1^ P. aeruginosa bacteria per nematode.

Because RNAi for *mul-1* results in delayed death upon P. aeruginosa infection (Fig. 1), we aimed to better understand the mechanism by which *mul-1* contributes to the enhanced pathogen resistance of the animals. We tested the ability of P. aeruginosa to colonize the nematode over the course of infection. RNAi for *mul-1* resulted in reduced accumulation of bacteria, determined by obtaining P. aeruginosa CFU counts from infected animals and measuring the fluorescence intensity of animals infected with P. aeruginosa expressing green fluorescent protein (GFP). Initially, over the first 4 h, RNAi *mul-1* animals accumulated P. aeruginosa at a rate similar to that of the control animals, but there were significantly fewer CFU recovered per nematode after 4 h (Fig. 2A), a trend that continued until at least 24 h postinfection (Fig. 2B). The remaining number of bacteria in the nematode may be the cause of death, although delayed, in the RNAi *mul-1* animals. There was little to no fluorescent signal found in RNAi *mul-1* animals in contrast to levels in RNAi control animals at 24 h postinfection (Fig. 2C), demonstrating that P. aeruginosa is not capable of successfully colonizing the intestine of RNAi *mul-1* animals to the same extent as the intestine of RNAi control animals. To determine if the enhanced resistance to infection is a general phenotype in the RNAi *mul-1* animals, we also exposed these animals to Salmonella enterica and monitored survival. We also observed that RNAi *mul-1* nematodes were more resistant to S. enterica infection than RNAi control nematodes (Fig. S1A), accumulated fewer S. enterica bacteria (Fig. S1B), and more rapidly cleared the infection (Fig. S1C). In addition to testing survival on S. enterica, we also tested for differences in survival rates between RNAi control and RNAi *mul-1* animals upon exposure to S. aureus (Fig. S1D). There were no differences in nematode survival rates under these conditions, suggesting that *mul-1* may play different roles in response to different pathogens.

10.1128/mBio.00060-20.1 FIG S1 (A) Young adult control and *mul-1* RNAi animals were exposed to S. enterica, and survival was monitored (3 biological, 3 technical replicates each; *n* = 180 animals). (B) Young adult control and *mul-1* RNAi animals were exposed S. enterica GFP. At indicated time points, CFU counts per nematode were determined. (C) Young adult control and *mul-1* RNAi animals were exposed S. enterica GFP for 24 h. At indicated time points after removal from S. enterica, CFU counts per nematode were determined. ***, *P* < 0.001; ****, *P* < 0.0001. (D) Young adult control and *mul-1* RNAi animals were exposed to S. aureus, and survival was monitored (3 biological, 3 technical replicates each, *n* = 180 animals). Download FIG S1, TIF file, 0.8 MB. Copyright © 2020 Hoffman et al. 2020 Hoffman et al. This content is distributed under the terms of the Creative Commons Attribution 4.0 International license.

**FIG 3:**
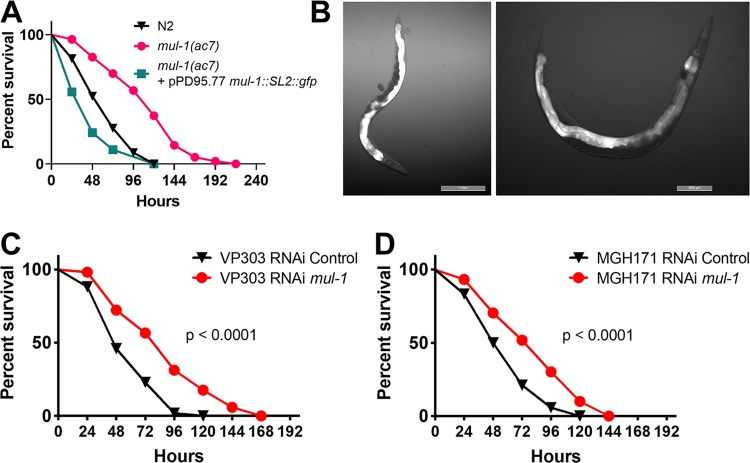
The enhanced-resistance phenotype observed in *mul-1*-silenced animals is intestine specific. (A) Young adult wild-type, *mul-1*(*ac7*), and *mul-1* rescue [*mul-1*(*ac7*)/ pPD95.77* mul-1*::*SL2*::GFP] animals were exposed to P. aeruginosa (3 biological and 3 technical replicates; *n* = 180 animals per condition). For results with wild-type versus *mul-1*(*ac7*) animals, *P* < 0.0001; for results with wild-type versus *mul-1*(*ac7*)/pPD95.77 *mul-1*::*SL2*::GFP animals, *P* < 0.001. (B) *mul-1* rescue [*mul-1*(*ac7*)/pPD95.77 *mul-1*::*SL2*::GFP] animals were imaged for cell-specific localization of *mul-1.* The GFP signal is located in the intestine. Representative images were selected of ∼30 observed animals. (C and D) Young adult and control and *mul-1* RNAi VP303 and MGH171 animals were exposed to P. aeruginosa (3 biological and 3 technical replicates; *n* = 180 animals per condition).

To determine whether RNAi for *mul-1* results in enhanced longevity, which could play a part in the observed enhanced resistance to P. aeruginosa, we examined the life span of RNAi control and *mul-1* animals on heat-killed nonpathogenic E. coli. As shown in Fig. S2A, the longevity of RNAi *mul-1* animals was indistinguishable from that of control animals, ruling out the possibility that enhanced longevity enhances survival in the presence of pathogenic bacteria. The *mul-1*(*ac7*) strain also showed significantly fewer CFU per nematode after 4 h of infection (Fig. 2D) and at 24 h (Fig. 2E). There was no difference in the life span of *mul-1*(*ac7*) nematodes in comparison to that of wild-type N2 nematodes (Fig. S2B). Rescue of *mul-1* under its own promoter in *mul-1* (*ac7*) nematodes resulted in increased susceptibility of the nematodes to P. aeruginosa infection (Fig. 3A). Several individual transgenic lines were tested for susceptibility to P. aeruginosa; all demonstrated enhanced susceptibility to the pathogen (Fig. S3A to E). We believe that the enhanced susceptibility to P. aeruginosa may be due to the overexpression of MUL-1, which may enhance P. aeruginosa colonization.

10.1128/mBio.00060-20.2 FIG S2 (A) After 2 generations of control or *mul-1* RNAi animals, L4 animals were transferred to fresh plates with heat-killed E. coli OP50. Survival was monitored daily (*n* = 180 animals per condition). (B) Larval stage 4 N2 wild-type and *mul-1*(*ac7*) animals were transferred to fresh plates of heat-killed E. coli OP50. Survival was monitored daily (*n* = 180 animals per condition). Download FIG S2, TIF file, 0.5 MB. Copyright © 2020 Hoffman et al. 2020 Hoffman et al. This content is distributed under the terms of the Creative Commons Attribution 4.0 International license.

10.1128/mBio.00060-20.3 FIG S3 Young adult N2 wild-type, *mul-1*(*ac7*) deletion animals, and *mul-1* rescue *mul-1*(*ac7*)*/*pPD95.77 *mul-1*::*SL2*::GFP animals were exposed to P. aeruginosa PA14. Survival was measured for each of the 5 individual transgenic lines (A to E) (3 biological, 3 technical replicates each; *n* = 180 animals). Download FIG S3, TIF file, 0.6 MB. Copyright © 2020 Hoffman et al. 2020 Hoffman et al. This content is distributed under the terms of the Creative Commons Attribution 4.0 International license.

Expression of *mul-1* was previously reported in the intestine, hypodermis, and PVD and OLL neurons (38, 39). In our study, we used the endogenous *mul-1* promoter (1.5 kb) and full *mul-1* gene to rescue gene expression in the *mul-1*(*ac7*) animals. Use of this *mul-1* rescue construct, which included a nontranslation fusion with GFP, demonstrated that the primary location of expression of *mul-1* is in the intestinal cells (Fig. 3B). This finding is consistent with the high levels of mRNA transcripts of *mul-1* found in the intestine (39). To address whether intestinal *mul-1* is specifically contributing to infection, intestine-specific RNAi was performed using two intestine-specific RNAi strains, VP303 (40) and MGH171 (41). In both cases, we found that RNAi *mul-1* in the intestine resulted in enhanced resistance to P. aeruginosa (Fig. 3C and D).

### Monosaccharides enhance P. aeruginosa killing of C. elegans.

Based on our data that show that *mul-1*(*ac7*) and RNAi *mul-1* animals are not colonized by P. aeruginosa, we hypothesized that MUL-1 plays a beneficial role for the pathogen. MUL-1 is predicted to have multiple glycosylation sites (Fig. S4), but the specific composition of the MUL-1 O-linked oligosaccharides is unknown. It has been demonstrated that O-linked glycans contain many of the same monosaccharides (d-galactose [Gal], d-glucose [Glc], *N*-acetyl-d-glucosamine [GluNAc], and *N*-acetyl-d-galactosamine [GalNAc]), but unusual features were identified. One of the identified mucin-type O-linked glycans had either type-1 core Galβ1,3GalNAc or one of three previously unidentified core types: (i) Galβ1,6(Galβ1,3)GalNAc, (ii) Glcβ1-6(Galβ1,3)GalNAc, or (iii) (Glcβ1-6[Glcβ1,4]Galβ1-3)GalNAc (42). The second mucin-type O-glycan identified in the study possessed a GlcNAc in the terminal position (42). Previous studies have shown that C. elegans O-glycans have common features shared with vertebrate glycans, but their terminal structures have differences. Indeed, C. elegans glycans lack sialic acid and instead contain O-methylated fucose and mannose as well as phosphorylcholine substitutions (43, 44). One of the major barriers experienced by pathogens in accessing monosaccharides present on mucins is the presence of sialic acid caps. In order to expose the host monosaccharides, bacteria require a sialidase enzyme, which removes the sialic acid caps, most often exposing a galactose residue (45–47). P. aeruginosa does not encode a bacterial sialidase (48) and relies on other sialidase-expressing bacteria present in the host to cleave sialic acids and access monosaccharides on glycans. Interestingly, this is not a barrier in C. elegans because the animals lack sialic acid caps, and thus P. aeruginosa can freely access monosaccharides.

10.1128/mBio.00060-20.4 FIG S4 Predicted glycosylation sites of the MUL-1 peptide sequence were determined using the GlycoPred software available through the Hirst Group website, maintained by Jonathan D. Hirst at the School of Chemistry at University of Nottingham (http://comp.chem.nottingham.ac.uk/glyco/. The prediction is made using random forests. Download FIG S4, TIF file, 2.0 MB. Copyright © 2020 Hoffman et al. 2020 Hoffman et al. This content is distributed under the terms of the Creative Commons Attribution 4.0 International license.

**FIG 4:**
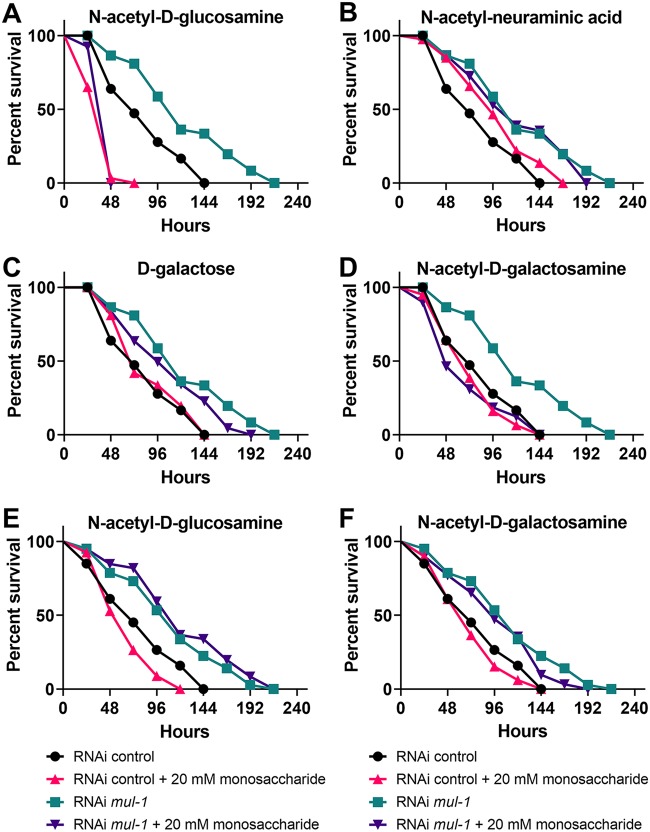
Monosaccharides alter the resistance phenotype of C. elegans to P. aeruginosa PA14. (A to D) Young adult control and *mul-1* RNAi animals were exposed to P. aeruginosa on plates containing the indicated monosaccharides at 20 mM. (A) Significance for results with *N*-acetyl-d-glucosamine was determined as follows: RNAi control versus RNAi *mul-1* animals, *P* < 0.0001; RNAi control versus *N*-acetyl-d-glucosamine RNAi *mul-1* animals, *P* <0.0001; RNAi control versus *N*-acetyl-d-glucosamine *mul-1* animals, not significant; RNAi *mul-1* versus *N*-acetyl-d-glucosamine RNAi *mul-1* animals, *P* < 0.0001. (B) Significance for results with *N*-acetyl-neuraminic acid was determined as follows: RNAi control versus RNAi *mul-1* animals, *P* < 0.0001; RNAi control versus *N*-acetyl-neuraminic acid RNAi control animals, *P* < 0.01; RNAi *mul-1* versus *N*-acetyl-neuraminic acid RNAi *mul-1* animals, not significant. (C). Significance for results with d-galactose was determined as follows: RNAi control versus RNAi *mul-1* animals, *P* < 0.0001; RNAi control versus d-galactose RNAi control animals, *P* < 0.01; RNAi *mul-1* versus d-galactose RNAi *mul-1* animals, not significant. (D) Significance for results with *N*-acetyl-d-galactosamine was determined as follows: RNAi control versus RNAi *mul-1* animals, *P* < 0.0001; RNAi control versus *N*-acetyl-d-galactosamine RNAi control animals, not significant; RNAi control versus *N*-acetyl-d-galactosamine RNAi *mul-1* animals, not significant; RNAi *mul-1* versus *N*-acetyl-d-galactosamine RNAi *mul-1* animals, *P* < 0.001. For the experiments shown in panels A to D, 3 biological and 3 technical replicates were used (*n* = 180 animals per condition). (E and F) Young adult control and *mul-1* RNAi animals were exposed to P. aeruginosa previously grown on 20 mM *N*-acetyl-d-glucosamine (RNAi control versus RNAi *mul-1* animals, *P* < 0.0001; RNAi control versus *N*-acetyl-d-glucosamine RNAi control animals, not significant; RNAi *mul-1* versus *N*-acetyl-d-glucosamine RNAi *mul-1* animals, not significant) or *N*-acetyl-d-galactosamine (RNAi control versus RNAi *mul-1* animals, *P* < 0.0001; RNAi control versus *N*-acetyl-d-galactosamine RNAi control animals, not significant; RNAi *mul-1* versus *N*-acetyl-d-galactosamine RNAi *mul-1* animals, not significant). For the experiments shown in panels E and F, 3 biological and 3 technical replicates were used (*n* = 180 animals per condition).

To better understand the role of MUL-1 inside the C. elegans intestine, we probed if monosaccharides present on mucins, including *N*-acetyl-d-galactosamine, galactose, *N*-acetyl-d-glucosamine, and *N*-acetylneuraminic acid, were important during P. aeruginosa infection. We tested individual monosaccharides for their effects on bacterial growth under a variety of *in vitro* growth conditions and found that within 24 h, the monosaccharides had no effect on P. aeruginosa growth (Fig. S5). This was to be expected because previous reports showed that *N-*acetyl-d-glucosamine does not enhance P. aeruginosa growth (49). Because the tested monosaccharides had no effect on bacterial growth, we investigated if monosaccharides could play a role during nematode infection. The addition of *N*-acetyl-d-glucosamine drastically decreased the survival of RNAi control and RNAi *mul-1* animals infected with P. aeruginosa (Fig. 4A). This may be due to the fact that *N-*acetyl-d-glucosamine had been previously reported to increase virulence factor production in P. aeruginosa (49). The *N-*acetyl-d-glucosamine has an effect on both the RNAi control and RNAi *mul-1* animals, likely because it is signaling for the increase in virulence gene expression in both animals. *N*-Acetylneuraminic acid (Fig. 4B) and d-galactose (Fig. 4C) had nonsignificant effects on survival of RNAi control or RNAi *mul-1* nematodes. *N*-Acetyl-d-galactosamine supplementation fully restored RNAi *mul-1* nematode survival to the level of control nematode survival on nonsupplemented plates while having no effect on RNAi control nematode survival (Fig. 4D), which suggests that P. aeruginosa may specifically require *N*-acetyl-d-galactosamine from MUL-1 during infection and that MUL-1 is the predominant source from which P. aeruginosa obtains *N*-acetyl-d-galactosamine. These results were unexpected and demonstrate the complexity of the mucosal barrier in which mucins, glycans, and monosaccharides play various roles during host-pathogen interactions.

10.1128/mBio.00060-20.5 FIG S5 P. aeruginosa growth at the OD_600_ in LB broth (A), M9 buffer (B), or (C) SK nematode-killing medium without agar in the presence of increasing concentrations of *N*-acetyl-d-glucosamine or *N*-acetyl-d-galactosamine or specified concentrations of *N*-acetylneuraminic acid or d-galactose. Bacteria were grown statically in 100-μL cultures in 96-well plates at 37°C (3 biological replicates and 3 technical replicates for each condition). Download FIG S5, TIF file, 0.7 MB. Copyright © 2020 Hoffman et al. 2020 Hoffman et al. This content is distributed under the terms of the Creative Commons Attribution 4.0 International license.

To determine whether P. aeruginosa must be exposed to the monosaccharides during infection or whether the monosaccharides alone cause an increase in virulence of the pathogen, bacteria were first cultured in the presence of monosaccharides at concentrations used in plates during the killing assays. Bacteria were then collected, washed, and used to seed full lawn plates on which survival for the RNAi control and RNAi *mul-1* nematodes was tested. Addition of *N*-acetyl-d-glucosamine (Fig. 4E) and *N*-acetyl-d-galactosamine (Fig. 4F) to the medium prior to seeding had no effect on nematode survival on P. aeruginosa lawns. These data suggest that monosaccharides are required to be present and accessible by the bacteria during infection for successful P. aeruginosa killing of the nematodes.

### Monosaccharides alter pyocyanin production and biofilm formation.

A recent study has shown that mucin-derived complex glycans attenuate the expression of P. aeruginosa virulence-related genes *in vitro*, reduce binding to cells, and reduce bacterial burden in a porcine burn model (3). It was also reported that a mixture of monosaccharides does not alter binding to host cells, bacterial aggregation, or the expression of virulence-related genes. Because our results in C. elegans indicate that a mucin, MUL-1, and monosaccharides are required for full P. aeruginosa pathogenesis in this system, we tested whether the individual monosaccharides that play a role in the C. elegans system may alter virulence traits of the bacteria, including pyocyanin production and biofilm formation.

**FIG 5:**
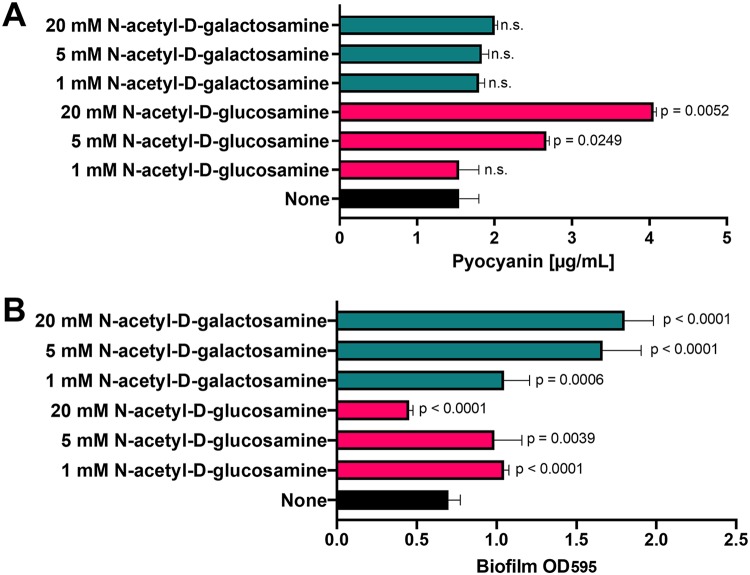
*N*-Acetyl-d-glucosamine increases pyocyanin production, and *N*-acetyl-d-galactosamine enhances biofilm formation. (A) Pyocyanin production was quantified using previously described methods. Bacterial culture supernatants were collected from bacteria grown in the absence or presence of increasing concentrations of *N*-acetyl-d-glucosamine and *N*-acetyl-d-galactosamine (3 biological replicates). *P* values are indicated on the figure (n.s., nonsignificant). (B) P. aeruginosa biofilm formation was measured in 96-well, static 100-μl cultures using a crystal violet assay. Bacteria were grown in the absence or presence of increasing concentrations of *N*-acetyl-d-glucosamine and *N*-acetyl-d-galactosamine (3 biological replicates and 3 technical replicates each). *P* values are indicated on the figure.

Phenazines, including pyocyanin, are pigmented, redox-active molecules secreted by P. aeruginosa and display a broad range of toxic activities toward both prokaryotic and eukaryotic organisms (28). In humans, pyocyanin is one of the major virulence factors that contributes to both chronic and acute infections (50). The toxin damages epithelial cells through hydroxyl radical formation (50, 51), inactivates protease inhibitors (52), and targets many cellular processes (53–56). In addition, it can be a marker for the upregulation of other virulence factors, including those associated with efflux and redox processes, as well as iron acquisition (57, 58). To determine whether monosaccharides have an effect on pyocyanin production, P. aeruginosa was grown statically in Luria-Bertani (LB) broth supplemented with increasing concentrations of *N-*acetyl-d-glucosamine and *N*-acetyl-d-galactosamine for 24 h, at which point supernatants were collected and used to quantify pyocyanin production (59, 60). *N-*Acetyl-d-glucosamine increased pyocyanin production in a concentration-dependent manner while *N*-acetyl-d-galactosamine had no effect on toxin production (Fig. 5A).

We also studied the ability of the two monosaccharides to alter biofilm formation *in vitro*. The medium was supplemented with increasing concentrations of *N-*acetyl-d-glucosamine or *N*-acetyl-d-galactosamine, and biofilm formation (bacterial attachment to an abiotic surface) was measured. *N-*Acetyl-d-glucosamine had little to no effect on biofilm formation, but *N*-acetyl-d-galactosamine enhanced P. aeruginosa biofilm formation in a concentration-dependent manner (Fig. 5B).

### Monosaccharides increase binding of P. aeruginosa to human lung epithelial cells.

**FIG 6:**
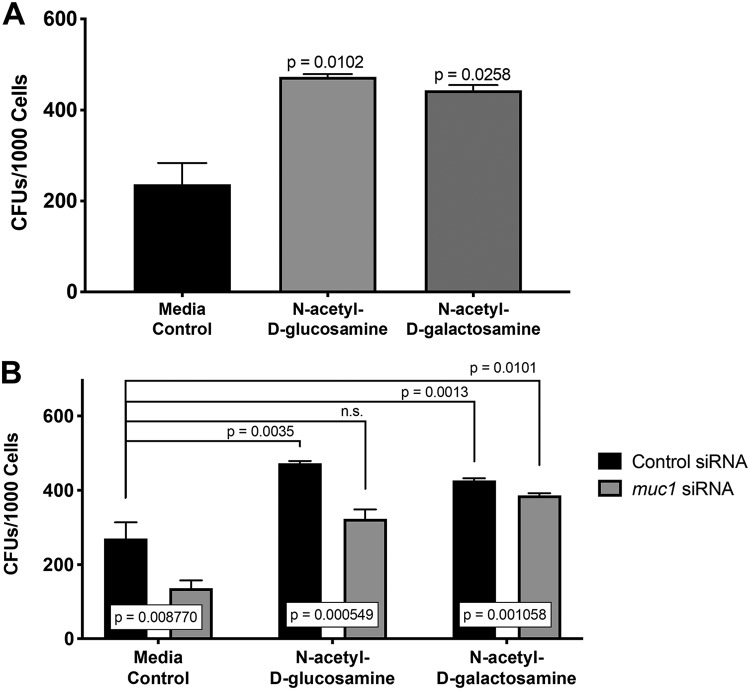
Monosaccharides alter binding of P. aeruginosa PA14 to A549 human lung cells. (A) *N-*Acetyl-d-glucosamine or *N*-acetyl-d-galactosamine at 2 mM was added to A549 lung cells with P. aeruginosa (MOI of 100) for 4 h. Total bacterial CFU counts (both bound extracellular and internal) were determined and normalized to the value for total viable cells (3 biological replicates and 3 technical replicates each). *P* values are indicated on the figure (n.s., nonsignificant). (B) Sigma Mission TRC control and *muc1* shRNA lentiviral particles were used to create control and *muc1* knockdown cells. *N-*Acetyl-d-glucosamine or *N*-acetyl-d-galactosamine at 2 mM was added to cells with P. aeruginosa (MOI of 100) for 4 h. Total bacterial CFU counts (both bound extracellular and internal) were determined and normalized to the value for total viable cells (3 biological replicates and 3 technical replicates each). *P* values are indicated on the figure.

Because mucins are conserved across species and because the aforementioned monosaccharides are also present in a range of organisms, we hypothesized that the individual monosaccharides identified in the C. elegans system would have similar effects on the binding of P. aeruginosa to human lung cells. A549 human lung alveolar epithelial cells were used to assess bacterial binding in the presence of monosaccharides. These cells have been previously shown to express only mucins MUC1 and MUC5a (61, 62). The use of this cell culture model system, with only two expressed mucins, would make it simpler to distinguish how individual mucins contribute to bacterial binding phenotypes. The A549 cells were seeded and grown for approximately 2 days, at which time 2 mM *N-*acetyl-d-glucosamine or *N*-acetyl-d-galactosamine was added to cells simultaneously with P. aeruginosa. The concentration of 2 mM monosaccharides and the 4-h time point were chosen because of the effects of monosaccharides on bacterial growth at later time points (Fig. S6). We found that both *N-*acetyl-d-glucosamine and *N*-acetyl-d-galactosamine significantly increased bacterial binding to cells compared to levels with medium alone (Fig. 6A).

10.1128/mBio.00060-20.6 FIG S6 P. aeruginosa growth at the OD_600_ in F12K medium –10% FBS in the presence of increasing concentrations *N*-acetyl-d-glucosamine (A) and *N*-acetyl-d-galactosamine (B). Bacteria were grown statically in 100-μL cultures in 96-well plates at 37°C (2 biological replicates, 6 technical replicates each). Download FIG S6, TIF file, 0.5 MB. Copyright © 2020 Hoffman et al. 2020 Hoffman et al. This content is distributed under the terms of the Creative Commons Attribution 4.0 International license.

We have shown that monosaccharides, specifically *N*-acetyl-d-galactosamine, reverse the enhanced resistance phenotype of RNAi *mul-1* nematodes. We then investigated if monosaccharides also reverse binding defects in human lung cells, which lack mucins required for bacterial binding. Short hairpin RNA (shRNA) silencing was used to knock down expression of *muc1* in A549 cells. As previously demonstrated (6, 61), we also show that knockdown of *muc1* reduced the number of P. aeruginosa bacteria bound per cell (Fig. 6B). This is similar to the observation that in C. elegans lacking *mul-1*, there is a decrease in the numbers of P. aeruginosa cells in the nematode, suggesting that mucins and monosaccharides may play similar roles in different models of bacterial pathogenesis. Consistent with this idea, when *N-*acetyl-d-glucosamine and *N*-acetyl-d-galactosamine were added to control cells, they enhanced bacterial binding (Fig. 6B). In addition, both monosaccharides also reversed the binding defect of P. aeruginosa to *muc1* shRNA knockdown cells, restoring the number of bacteria per cell to that of control cells in medium alone.

## DISCUSSION

This work demonstrates the ability of the pathogen P. aeruginosa to exploit a host mucin to successfully colonize mucosal epithelial barriers and infect a host. Our data specifically show that silencing or deleting the C. elegans pathogen-induced immune effector *mul-1* results in enhanced resistance to P. aeruginosa. Although the exact manner in which P. aeruginosa requires MUL-1 remains unknown, it appears that P. aeruginosa exploits individual monosaccharides from MUL-1. This is substantiated by the findings indicating that the enhanced resistance to P. aeruginosa of RNAi *mul-1* animals is reversed by the addition of *N*-acetyl-d-galactosamine. MUL-1 may serve as a binding site for P. aeruginosa, provide a carbon source for growth *in vivo*, or serve as a cue to alter the virulence of the bacteria.

Mucins and the monosaccharides that comprise O-linked glycans are conserved among many organisms. It had been previously reported that P. aeruginosa binding to human lung epithelial cells is reduced in the absence of specific mucins, namely, MUC1 (6, 14, 17, 61). In addition, we have also demonstrated that monosaccharides reversed the binding defects observed in *muc1*-silenced cells, indicating that mucins may aid bacterial pathogenesis. A recent study reported that mucin-derived glycans attenuate P. aeruginosa virulence but that monosaccharides do not (3). In this aforementioned study, P. aeruginosa PAO1, intestinal mucus, and mixtures of monosaccharides were used. Our data show that unlike glycans and mixtures of monosaccharides, individual monosaccharides are able to enhance virulence traits of P. aeruginosa both in C. elegans and in human cell lines. This suggests that bacteria may be able to break down glycans that attenuate virulence to access individual monosaccharides, which may serve as a mechanism to increase pathogenesis. It appears that mucins, their glycans, and monosaccharides play a diverse set of roles during infection and that these host factors can impact virulence. It is possible that the repertoire of glycans and monosaccharides, which varies throughout the body, may contribute to differences in bacterial binding to various tissues as well.

The ability to control a pathogen’s access to mucins, glycans, and the derived monosaccharides could prove to be a method to prevent, control, and/or disrupt infection. In certain disease states, opportunistic pathogens are able to colonize and establish an infection when there are changes in the levels of mucus. In some cases, the glycosylation patterns of mucins change, and this results in enhanced binding of pathogens to the epithelial cells. Therapeutics that control mucin expression, prevent binding of pathogens to mucins, or alter bacterial virulence would be extremely beneficial in treating and preventing disease and opportunistic pathogen infections.

In summary, we have identified the host mucin, MUL-1, in C. elegans as an innate immune factor that is exploited by P. aeruginosa during infection. P. aeruginosa likely acquires monosaccharides from the mucin during infection to enhance pathogenesis and rapidly kill the nematode. The same monosaccharides identified to enhance virulence in C. elegans also enhance P. aeruginosa binding in human lung cells, suggesting a conserved host factor at mucosal barriers that alters pathogen infection. This idea that bacteria access monosaccharides from mucins would require bacteria to express a sialidase enzyme to cleave sialic acid from oligosaccharides to access individual monosaccharides in mammalian systems. P. aeruginosa does not encode a sialidase but can rely upon sialidases from other bacteria present in the host to access the monosaccharides (63). It is possible that members of the microbiome produce sialidases that cleave sialic acids, exposing monosaccharides that are more easily utilized by P. aeruginosa. Understanding the mechanisms by which other bacteria may aid P. aeruginosa to obtain resources from mucins may provide interesting insights into the understanding of polymicrobial infections and the roles of the microbiota in disease.

## MATERIALS AND METHODS

### Bacterial strains.

The following bacterial strains were used: Escherichia coli OP50 (from the *Caenorhabditis* Genetics Center [CGC]), E. coli HT115 (DE3) (from OpenBioSource), Pseudomonas aeruginosa PA14, P. aeruginosa PA14 expressing enhanced green fluorescent protein (PA14-EGFP) (Amp^r^, Kan^r^) (64), Salmonella enterica serovar Typhimurium 1344 (Sm^r^) (65), and Salmonella enterica serovar Typhimurium-EGFP SM022 (Sm^r^, Kan^r^) (66). All strains were maintained on Luria-Bertani (LB) agarose plates with required antibiotics or LB broth with required antibiotics at 37°C shaking at 250 rpm.

### C. elegans strains and growth conditions.

C. elegans hermaphrodites were maintained on E. coli OP50 at 20°C unless otherwise indicated. Bristol N2 was used as the wild-type control strain. The following were used: PHX1027 *mul-1* (*syb1027*) IV, a CRISPR/Cas9 ∼1,650-bp deletion mutant of isoform A and B (565 bp and 952 bp deleted, with generated termination codon), with a predicted truncated protein of 46 amino acids (generated by SUNY Biotech); AY157 *mul-1*(*ac7*) IV; PHX1027 *mul-1* (*syb1027*) backcrossed six times with N2; AY158 *mul-1*(*ac7*) IV [pPD95.77* mul-1p*::*mul-1*::*SL2*::GFP]. The Fire Lab C. elegans vector kit was a gift from Andrew Fire (1000000001; Addgene). Gut-sensitive RNAi lines were the following: MGH171 *sid-1* (*qt9*) V; alxIs9 (*alxIs9* [*vha-6p*::*sid-1*::*SL2*::GFP]); VP303 *rde-1* (*ne219*) V; kbIs7 (*kbIs7* [*nhx-2p*::*rde-1 rol-6* (*su1006*)]) (from the *Caenorhabditis* Genetics Center, University of Minnesota, Minneapolis, MN).

### RNAi.

RNAi interference (RNAi) was used to generate loss-of-function RNAi phenotypes by feeding nematodes E. coli strain HT115 (DE3) expressing double-stranded RNA (dsRNA) homologous to a target gene (67–69). RNAi was carried out as described previously (70). Briefly, E. coli with appropriate vectors was grown in LB broth containing ampicillin (100 μg/ml) and tetracycline (12.5 μg/ml) at 37°C overnight and plated onto nematode growth medium (NGM) plates containing 100 mg/ml ampicillin and 6 mM isopropyl-β-d-thiogalactoside (IPTG) (RNAi plates). RNAi-expressing bacteria were allowed to grow overnight at 37°C. Gravid adults were transferred to RNAi-expressing bacterial lawns and allowed to lay eggs for 8 h. Two generations of RNAi were performed. RNAi targeting *unc-22* was included as a positive control to account for the RNAi efficiency. Because RNAi for *let-653* is larval lethal, RNAi was performed on stage 4 larvae (L4). All RNAi clones except *mul-1* were from the Ahringer RNAi library (Open BioSource). The *mul-1* clone was obtained from the Vidal RNAi library (Open BioSource). All clones were sequenced using universal GeneWiz M13 primers.

### Killing assay and survival on pathogens.

Bacterial lawns were prepared by inoculating individual bacterial colonies into 2 ml of LB broth with 50 mg/ml kanamycin and growing them for 7 to 8 h on a shaker at 37°C. For the colonization assays, bacterial lawns of P. aeruginosa or S. enterica serovar Typhimurium were prepared by spreading 35 μl of the culture over the complete surface of 3.5-cm-diameter modified NGM agar plates (3.5% instead of 2.5% peptone). Young adult animals were transferred to full lawns of P. aeruginosa PA14 or S. enterica serovar Typhimurium, and nematode survival was monitored daily. A total of 180 animals per condition were used unless otherwise indicated. Animals were considered dead upon failure to respond to touch. Animals missing from the agar plate were censored on the day of loss. The Kaplan-Meier method was used to calculate the survival fractions, and statistical significance between survival curves was determined using a log rank test.

### P. aeruginosa-GFP colonization assay.

Colonization assays were performed as previously described (71). Briefly, bacterial lawns were prepared by inoculating individual bacterial colonies into 2 ml of LB broth with 50 mg/ml kanamycin and growing them for 7 to 8 h on a shaker at 37°C. For the colonization assays, bacterial lawns of P. aeruginosa-GFP were prepared by spreading 35 μl of the culture over the complete surface of 3.5-cm-diameter modified NGM agar plates (3.5% instead of 2.5% peptone). The plates were incubated at 37°C for 12 to 16 h and then cooled to room temperature for at least 1 h before being seeded with young gravid adult hermaphroditic animals. The assays were performed at 25°C. At 24 hours, the animals were transferred from P. aeruginosa-GFP plates to fresh E. coli OP50 plates and visualized within 5 min under a fluorescence microscope.

### Quantification of intestinal bacterial loads.

P. aeruginosa-GFP lawns were prepared as described above. For quantification of CFU at various time points, bacterial lawns of P. aeruginosa-GFP were prepared by spreading 35 μl of the culture over the complete surface of 3.5-cm-diameter modified NGM agar plates (3.5% instead of 2.5% peptone). The plates were incubated at 37°C for 12 to 16 h and then cooled to room temperature for at least 1 h before being seeded with young adult hermaphroditic animals. The assays were performed at 25°C. At 1, 2, 3, 4, 5, 6, 7, 8, and 24 hours, the animals were transferred *from* P. aeruginosa-GFP plates to the center of fresh E. coli plates for 30 min to eliminate P. aeruginosa-GFP bacteria stuck to their bodies. Animals were transferred again to the center of a new E. coli plate for 30 additional minutes to further eliminate external P. aeruginosa-GFP bacteria. A total of 90 animals per condition were used, but the grinding was performed using 10 animals at a time. Ten animals were transferred into 50 μl of PBS plus 0.01% Triton X-100 and ground using glass beads. Serial dilutions of the lysates (10^−1^, 10^−2^, 10^−3^, 10^−4^, and 10^−5^) were seeded onto LB agar plates containing 50 mg/ml of kanamycin to select for P. aeruginosa-GFP cells. Plates were incubated overnight at 37°C. Single colonies were counted the following day, and results are represented as the number of bacterial cells or CFU per animal. Three independent experiments were performed for each condition.

### Fluorescence imaging.

Fluorescence imaging was carried out as described previously (70). Briefly, animals were anesthetized using an M9 salt solution containing 30 mM sodium azide and mounted onto 2% agar pads. The animals were then visualized using a Leica M165 FC fluorescence stereomicroscope.

### C. elegans longevity assays and cultivation of C. elegans on heat-killed E. coli OP50.

A single colony of E. coli OP50 was inoculated in 100 ml of LB broth in a 500-ml Erlenmeyer flask and incubated at 37°C with shaking at 225 rpm for 24 h. Bacteria were concentrated 20 times and heat killed at 100°C for 1 h. Bacterial death was confirmed by failure of the bacteria to grow on LB agar plates at 37°C overnight. The concentrated, heat-killed bacteria were seeded on NGM plates containing 50 mg/ml of kanamycin and 100 mg/ml of streptomycin. Young adult wild-type N2 animals grown on E. coli HT115 RNAi control or target gene RNAi plates were washed with M9 medium and incubated at room temperature for 1 h with M9 medium containing 50 mg/ml of kanamycin to remove live bacteria from the intestinal lumen. The animals were then washed with M9 medium and transferred to NGM plates containing heat-killed E. coli OP50 and incubated at 20°C for the duration of the assay. Remaining animals were transferred when the heat-killed E. coli OP50 lawn was reduced.

### Bacterial growth assays.

Individual bacterial colonies were inoculated into 2 ml of LB broth and grown overnight at 37°C with shaking at 225 rpm. Overnight cultures were diluted to an optical density at 570 nm (OD_570_) of 0.05 in either LB broth medium, M9 liquid medium, or Dulbecco’s modified Eagle’s medium-Ham’s F-12 (Kaighn’s) medium (DMEM/F12K) plus 10% heat-inactivated fetal bovine serum (FBS). Each of the described diluted bacterial cultures (100 μl) were placed in individual wells of a 96-well plate, with various monosaccharides added at indicated concentrations. Bacterial growth was monitored over time by measuring the OD_570_ at indicated time points.

### Measurement of pyocyanin production.

Pyocyanin was quantified using previously described methods (59, 60). Briefly, pyocyanin was extracted from cell-free bacterial supernatant after centrifugation at 10,000 rpm for 10 min and filtered. The pyocyanin concentration was quantified after measurement of the pyocyanin absorbance in the acidic form at 520 nm, according to the following equation: concentration of pyocyanin (in micrograms/milliliter)/ml = OD_520_ × 17.072.

### Measurement of biofilm formation.

P. aeruginosa biofilm was measured using a microtiter plate assay, coupled with crystal violet (CV) staining, as previously described (72, 73). Briefly, bacteria were grown in a total volume of 100 μl of LB broth at 37°C in 96‐well polyvinylchloride (PVC), round‐bottom, non‐tissue‐culture-treated microtiter plates. Biofilm was measured at 6 h. Wells were washed at the final time point to remove planktonic bacteria. Bacterial cells that remained attached to the wells were stained with a 0.1% solution of CV and incubated at room temperature for 30 min. The washing process was repeated, and the CV stain was solubilized from bacterial cells with 200 μl of 95% ethanol. Biofilm formation was quantitated by measuring the OD_595_.

### Cell lines.

The A549 human type II alveolar epithelial cell line (ATCC CCL-185; provided by David Lewinsohn’s lab at Oregon Health and Science University), at passage numbers 3 to 10, was used. A549 cells were maintained in Ham’s F12K (Kaighn’s) medium (21127022; Gibco) supplemented with 10% heat-inactivated fetal bovine serum (SAFC Biosciences, Lenexa, KS) without antibiotics. Cells were grown at 37°C with 5% CO_2_ and seeded every 4 days when confluence was approximately 85%.

### Lentiviral transduction with Mission TRC shRNA lentivirus particles.

The Mission TRC short hairpin RNA (shRNA) lentiviral transfection protocol was used. A total of 1 × 10^6^ A549 cells was plated in 60-mm dishes and grown for 20 h at 37°C in 5% CO_2_ to ∼60 to 70% confluence. Medium was replaced without 8 μg/ml hexadimethrine bromide. A multiplicity of infection (MOI) of ∼0.5 (∼13 μl of stock lentivirus) was added to the plates, and samples were incubated for 6 h. Medium was replaced without antibiotics for 24 h. The following day, medium was replaced with medium containing 5 μg/ml puromycin. Medium was replaced with fresh puromycin-containing medium every 3 to 4 days until resistant colonies grew to confluence. Cells were then used for the quantification of P. aeruginosa binding to control and shRNA cells, as described below.

### Quantification of bacterial binding to cells.

A gentamicin protection assay protocol was used as previously described (74), with some modifications (75). A549 cells were grown in 96-well plates, with or without monosaccharides present, for ∼2 days at 37°C in 5% CO_2_ until they reached approximately 85% confluence, corresponding to 1 × 10^5^ cells per well. Wells were washed four times with sterile PBS and serum-DMEM/F12 was added for 2 h. P. aeruginosa PA14 was grown overnight in LB broth at 37°C with shaking, diluted 1:10, and allowed to grow under the same conditions for 4 h (obtaining mid-log-phase bacteria). A549 cells were infected with P. aeruginosa PA14 at an MOI of 100 and incubated for 2 h at 37°C in 5% CO_2_. From the original bacterial suspension, serial dilutions were plated to verify the starting concentration of P. aeruginosa. Following incubation, the supernatants were removed, the wells were washed four times with PBS, and the numbers of associated and internalized bacteria were assessed as follows: 30 μl of 0.05% trypsin-EDTA was added to each well and incubated for 2 min at 37°C; cells were lysed with 70 μl of 0.1% Triton X-100 for 2 min at 37°C; lysates were removed, and serial dilutions were plated. Cell viability was measured using Promega CellTiter Glo 2.0 reagent (quantitates cellular ATP levels), and CFU counts were normalized per cell under each condition. These results represent the number of both attached and intracellular bacteria (number of CFU/number of cells).

### Quantification and statistical analysis.

Statistical analysis was performed with Prism, version 7 (GraphPad). All error bars represent the standard deviations (SD). A two-sample *t* test was used when needed, and the data were judged to be statistically significant at a *P* value of <0.05. The Kaplan-Meier method was used to calculate the survival fractions, and statistical significance between survival curves was determined using a log rank test. All experiments were performed in triplicate.
