# Supplementary material for: Host Mucin Is Exploited by Pseudomonas aeruginosa To Provide Monosaccharides Required for a Successful Infection
Source: mBio. 2020 Mar 3;11(2):e00060-20. doi: 10.1128/mBio.00060-20 (PMC7064748; doi:10.1128/mBio.00060-20)
Supplement: TABLE S1 [file mBio.00060-20-st001.pdf]

Gene	Sequence	Gene Details	Link to Infection	Associated Phenotypes
let-653	C29E6.1	mucin-like protein similar to highly glycosylated mucins of the apical surface of epithelia	Increase with *P. aeruginosa* infection	Larval lethal
gpdh-1	F47G4.3	encodes enzyme glycerol 3-phosphate dehydrogenase	Increase with *P. aeruginosa* infection	Human ortholog associated with Brugada Syndrome 2
gly-8	Y66A7A.6	encodes predicted transmembrane polypeptide N-acetylgalactosaminyl transferase (ppGaNTase)	Increase with *P. aeruginosa* infection	none
mul-1	F49F1.6	encodes mucin-like protein containing signal sequence and several ShK toxin domains	Increase with *P. aeruginosa* infection	Increased upon radiation exposure
cwp-4	K11D12.1	encodes a protein with similarity to mucins, predicted to be secreted	Increase with *P. aeruginosa* infection	Higher expression in males. Increased upon NaCl exposure
