# Supplementary figures and images for: Host Mucin Is Exploited by Pseudomonas aeruginosa To Provide Monosaccharides Required for a Successful Infection
Source: mBio. 2020 Mar 3;11(2):e00060-20. doi: 10.1128/mBio.00060-20 (PMC7064748; doi:10.1128/mBio.00060-20)

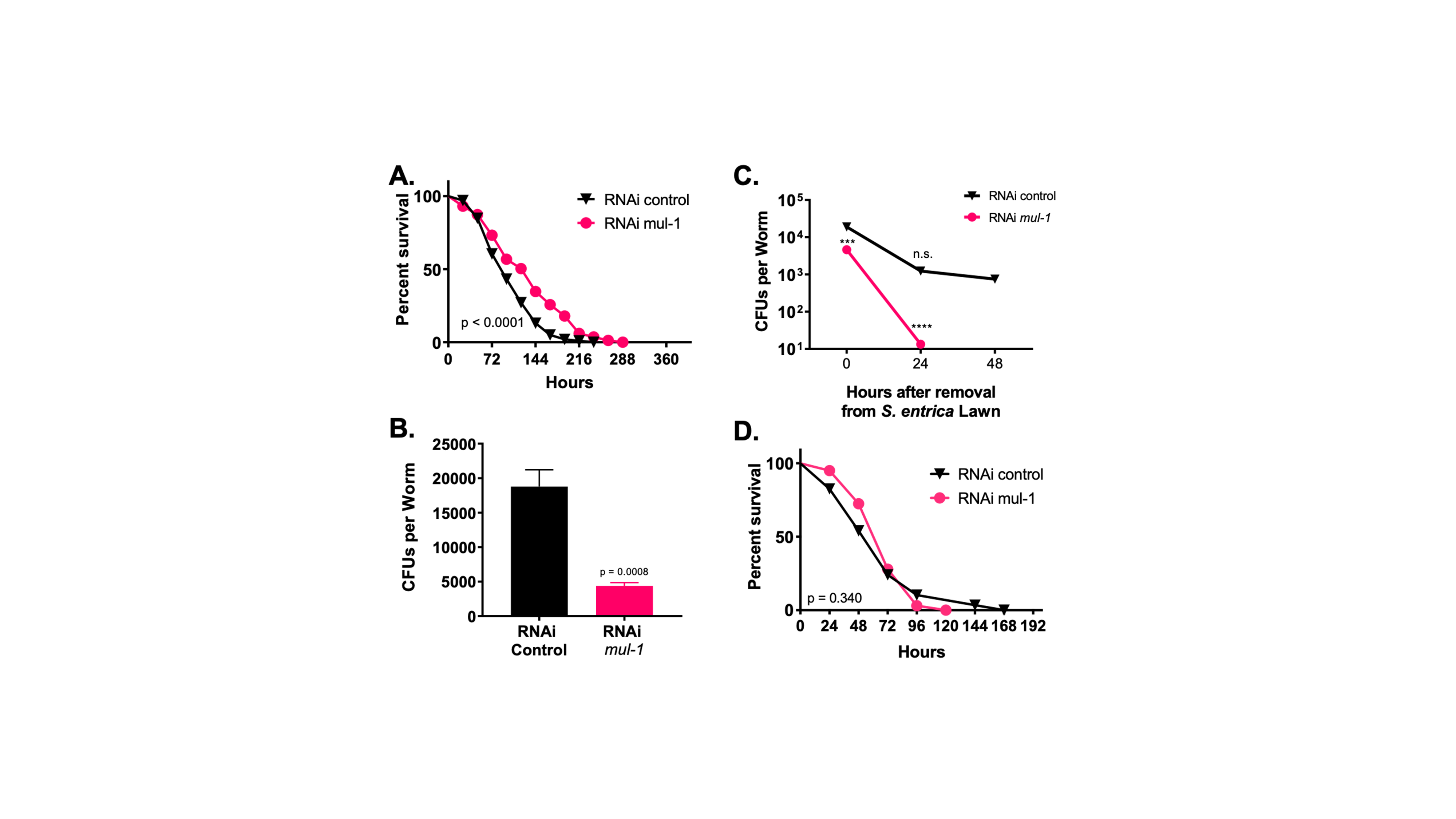

Supplement: FIG S1 [file mBio.00060-20-sf001.tif]

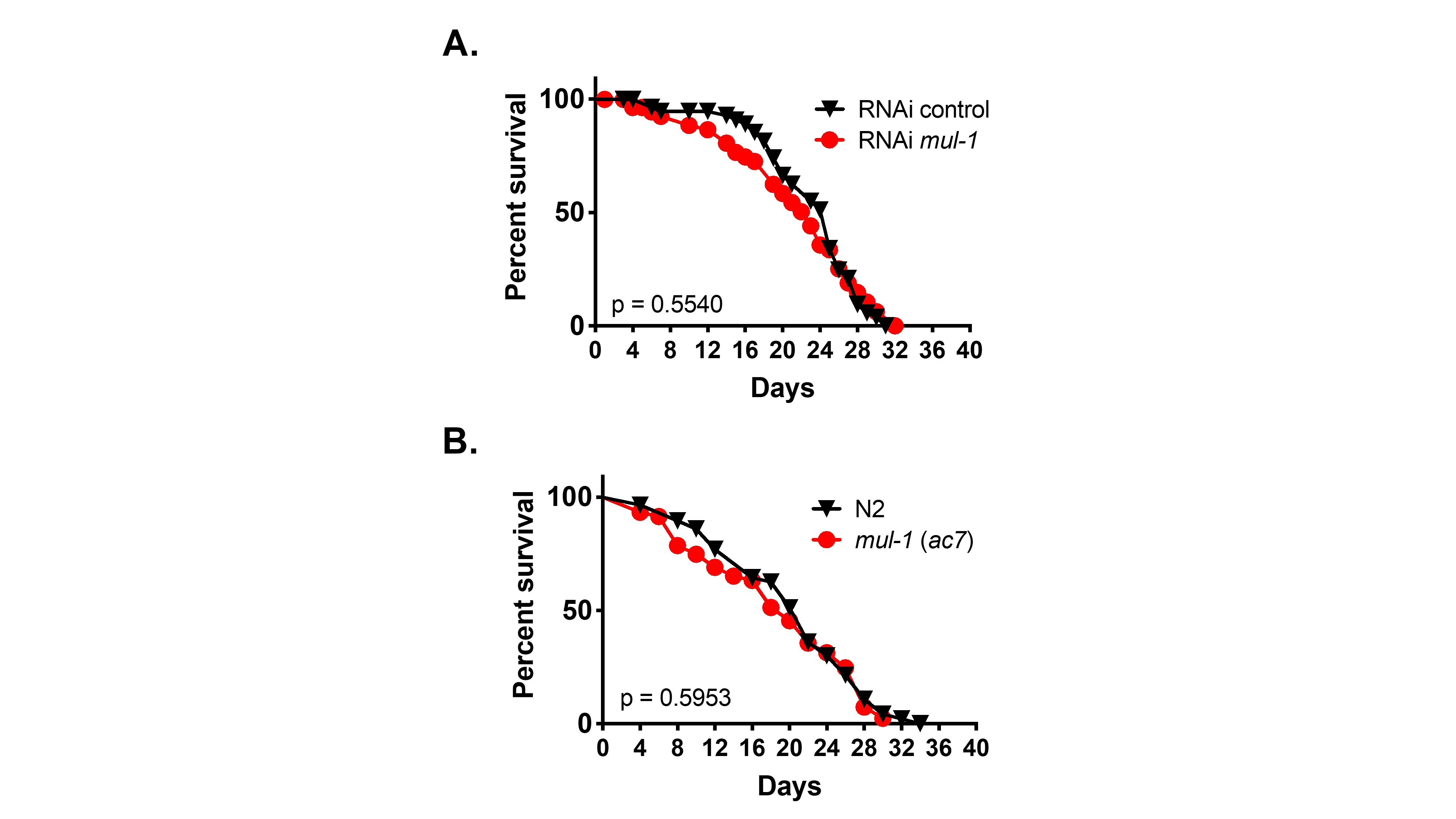

Supplement: FIG S2 [file mBio.00060-20-sf002.tif]

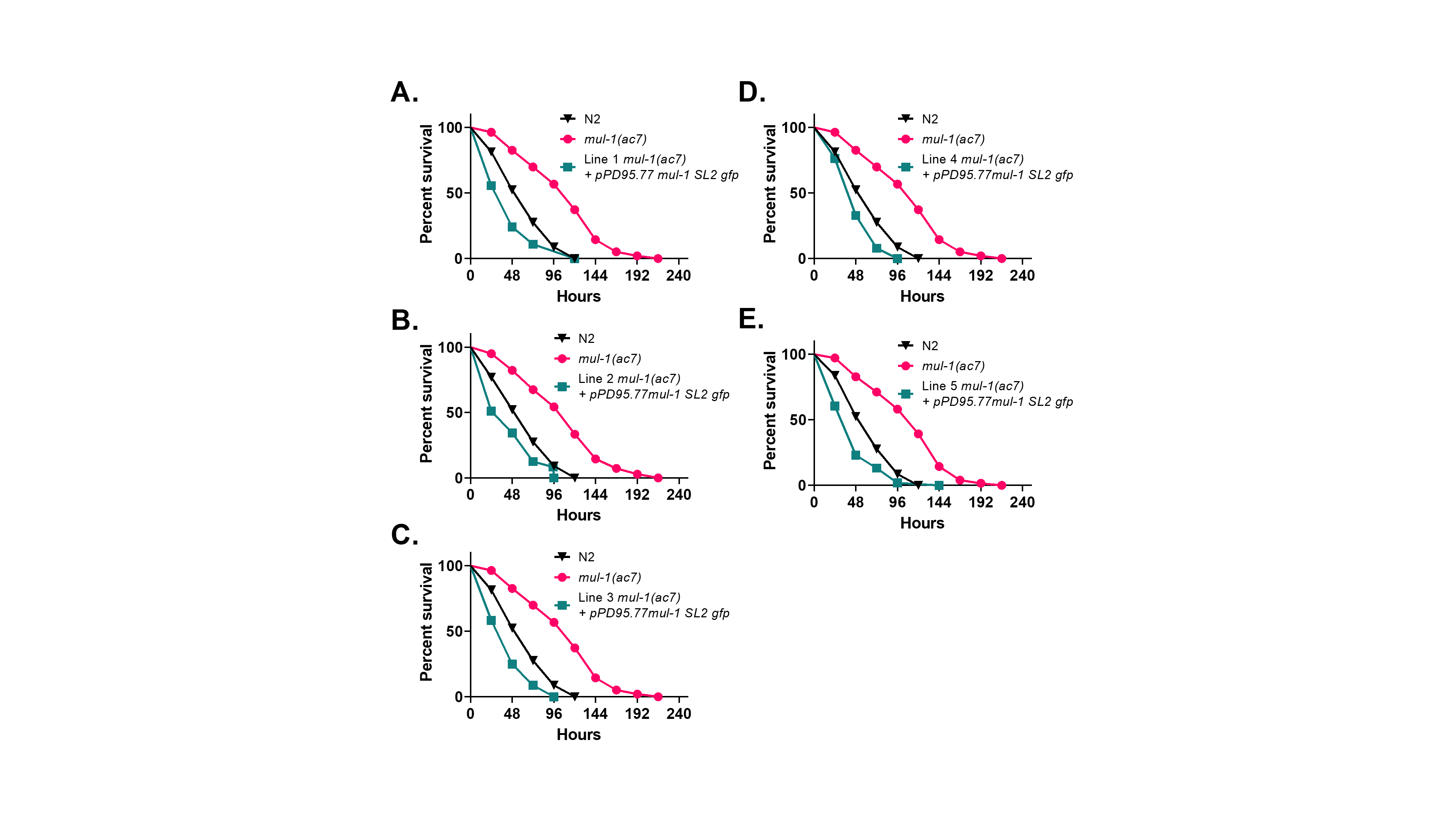

Supplement: FIG S3 [file mBio.00060-20-sf003.tif]

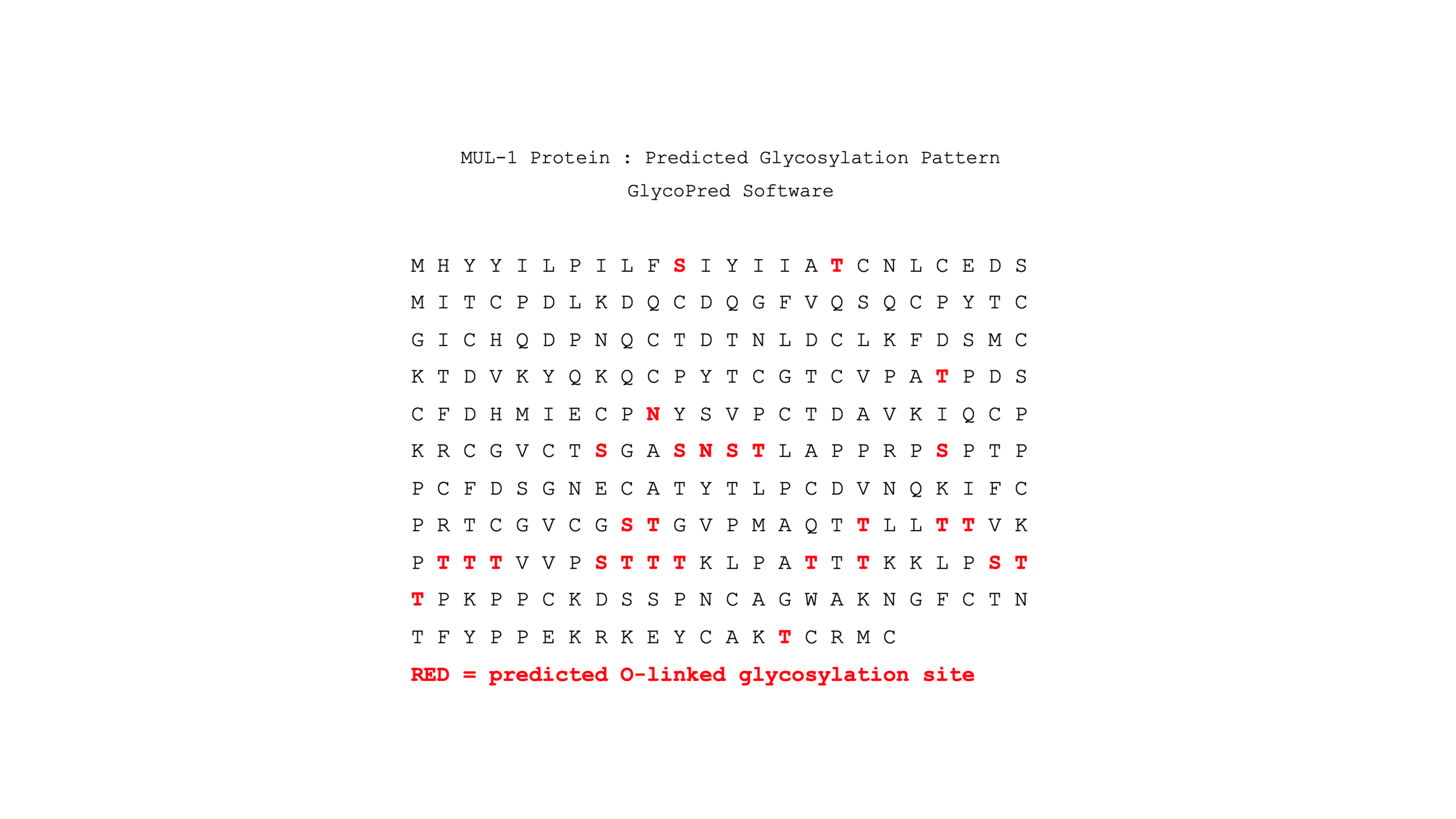

Supplement: FIG S4 [file mBio.00060-20-sf004.tif]

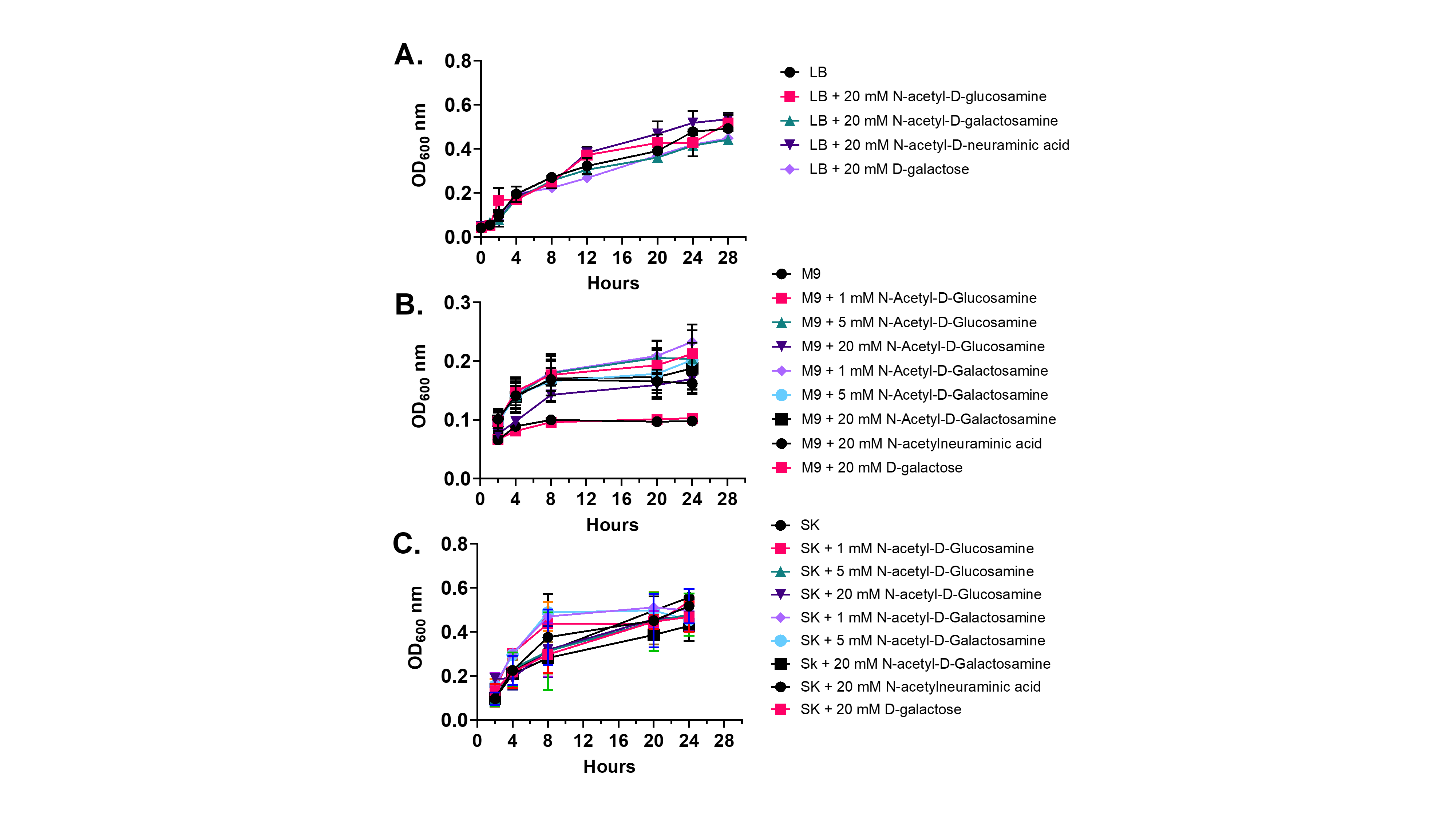

Supplement: FIG S5 [file mBio.00060-20-sf005.tif]

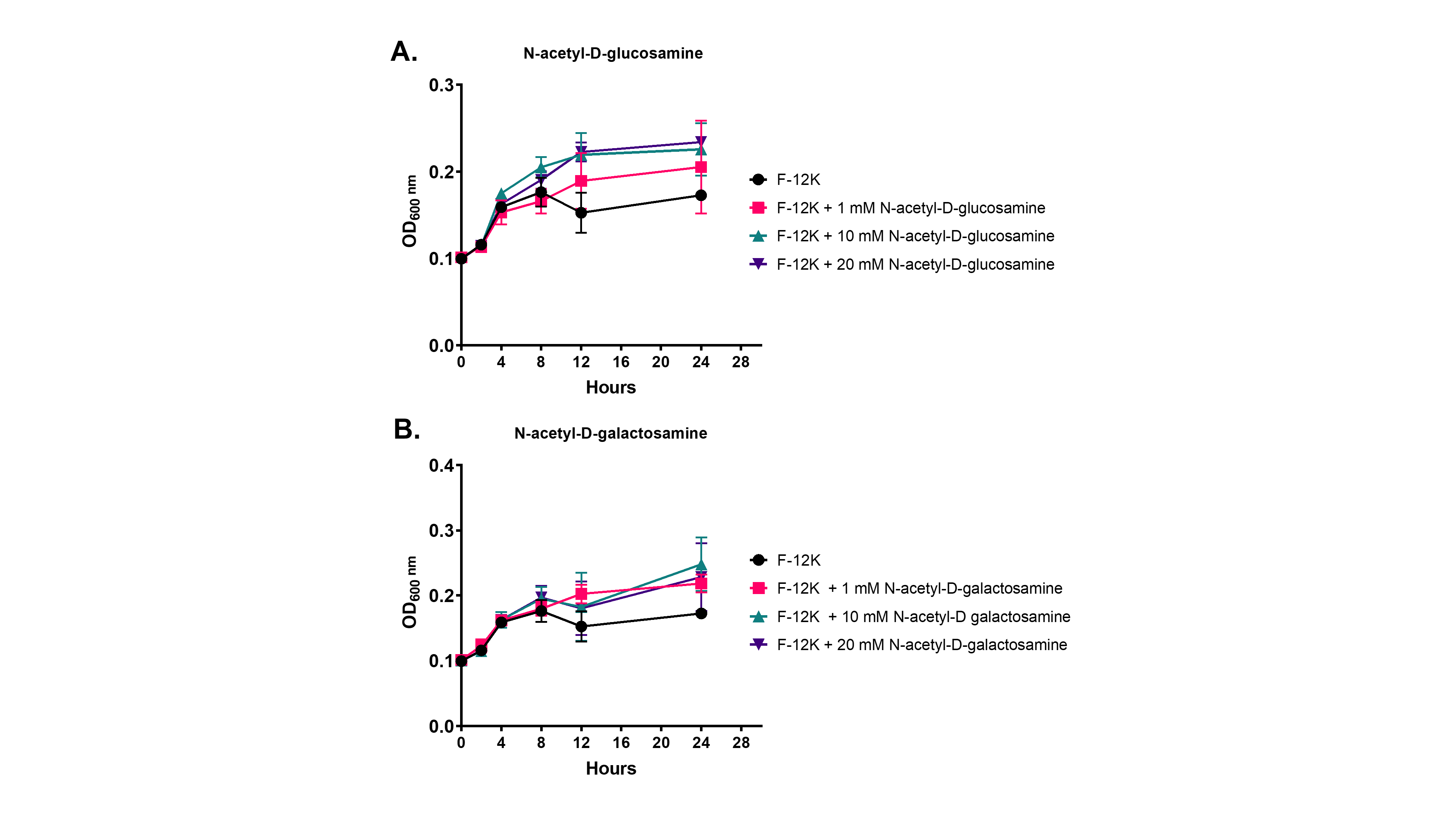

Supplement: FIG S6 [file mBio.00060-20-sf006.tif]
